# Physical and online food outlet availability and its influence on out-of-home dietary behaviours in Great Britain: A repeated cross-sectional study

**DOI:** 10.1016/j.ssmph.2025.101773

**Published:** 2025-03-05

**Authors:** Jody C. Hoenink, Yuru Huang, Matthew Keeble, Joreintje D. Mackenbach, Maria G.M. de Pinho, Lana Vanderlee, David Hammond, Christine M. White, Thomas Burgoine, Jean Adams

**Affiliations:** a MRC Epidemiology Unit, University of Cambridge School of Clinical Medicine, Box 285 Institute of Metabolic Science, Cambridge Biomedical Campus, Cambridge, CB2 0QQ, United Kingdom; b Upstream Team, www.upstreamteam.nl, Amsterdam UMC, the Netherlands; c Department of Marketing, Faculty of Business and Economics, University of Antwerp, Antwerp, Belgium; d Amsterdam UMC Location Vrije Universiteit Amsterdam, Epidemiology and Data Science, De Boelelaan 1117, Amsterdam, the Netherlands; e Amsterdam Public Health, Amsterdam, the Netherlands; f Copernicus Institute of Sustainable Development, Department Environmental Sciences, Utrecht University, Utrecht, the Netherlands; g Université Laval, Québec, QC, Canada; h School of Public Health Sciences, University of Waterloo, 200 University Avenue West, Waterloo, ON, N2L 3G1, Canada

**Keywords:** Digital foodscape, Fast food, Fast-food, Takeaways

## Abstract

**Background:**

As online food delivery service (OFDS) platforms gain popularity, understanding their impact on diet alongside physical food outlets is important for addressing suboptimal dietary quality. This study examined the independent and combined associations between physical and online food outlet availability and out-of-home dietary behaviours in 2019 and 2022. We also explored whether associations between physical outlet availability and dietary behaviours are modified by online food outlet availability.

**Methods:**

In this repeated cross-sectional analysis, we used British data from the adult International Food Policy Study (IFPS) in 2019 (n = 2912) and 2022 (n = 3544). Postcodes were used to assess neighbourhood food outlet availability using Ordnance Survey data and to determine OFDS availability on three platforms through web scraping. Associations were examined between neighbourhood outlet and OFDS availability with self-reported frequency of physical food outlet use, online food outlet use, and consuming meals prepared out-of-home.

**Results:**

In 2019 and 2022, both neighbourhood and OFDS availability were positively associated with all outcome measures. In 2019, after mutual adjustment, both availability measures remained associated with online food outlet use and consuming meals prepared out-of-home. However, in 2022, only OFDS availability was associated with these outcomes. For example, a one standard deviation increase in OFDS availability was associated with a 9% (95%CI 3%–14%) increase in frequency of consuming meals prepared out-of-home after adjusting for neighbourhood outlet availability. OFDS availability also modified associations between neighbourhood outlets and both online food outlet use and out-of-home meal consumption. As OFDS availability increased, the link between neighbourhood outlets and out-of-home meal consumption weakened.

**Conclusion:**

Neighbourhood outlet availability may influence out-of-home dietary behaviours, but its impact appears to weaken when OFDS availability is considered. Public health strategies should address the growing influence of OFDS platforms to improve dietary quality.

## Highlights

• Online food outlet use increased from 16% in 2019 to 25% in 2022 in Great Britain.
• Online Food Delivery Service (OFDS) availability is linked to greater out-of-home meal consumption.
• The link between neighbourhood outlet availability and dietary behaviours diminishes when adjusting for OFDS availability.
• Public health strategies should consider the online food environment.

## 1 Background

Exposure to the food environment, including the availability, accessibility, affordability and marketing of foods, can impact dietary quality (Boyland et al., 2016; Epstein et al., 2012). In turn, diet quality is associated with non-communicable diseases (Afshin et al., 2019). For example, greater consumption of takeaway food is associated with greater risk of type 2 diabetes, metabolic syndrome and cardiovascular disease (Bahadoran et al., 2016). Given the positive association between less healthy diets and the prevalence of non-communicable diseases, it is important to understand the relationship between the food environment and dietary behaviours. This understanding can inform the development of targeted strategies to improve aspects of public health, such as reducing obesity rates, lowering the burden of diet-related diseases, and increasing the availability of healthier foods.

The links between the availability and proximity of food outlets and their impact on dietary behaviours and body weight have been extensively studied (Caspi et al., 2012; E. Wilkins et al., 2019). For example, several studies from the United Kingdom (UK) found that greater takeaway (“fast-“) food outlet availability in residential neighbourhoods was associated with greater takeaway consumption and higher body weight (Burgoine et al., 2014; Burgoine et al., 2018; Vogel et al., 2017). However, these findings have not been consistently replicated in other studies either in the UK or elsewhere (Hickson et al., 2011; Mackenbach et al., 2017; Pinho et al., 2019). The increasing popularity of online food delivery service (OFDS) platforms like Deliveroo and Just Eat has underscored the necessity to expand our understanding of the food environment's influence on dietary behaviours beyond physical availability (Hoenink, Huang, et al., 2023).

OFDS platforms have expanded the availability of food prepared out-of-home (OOH), extending the reach of physical outlets through delivery services. Research indicates that the average delivery distances in Canada, New Zealand, and Australia ranges between 3.0 and 3.7 km (Brar & Minaker, 2021; Partridge et al., 2020), allowing these platforms to offer food options beyond what is locally available in residential (home) neighbourhoods. Importantly, online food availability is not merely an extension of physical availability; some online outlets, such as ‘dark kitchens' (delivery-only commercial kitchens), exist solely to meet OFDS demand without any physical presence (Da Cunha et al., 2024). The availability of OFDS platforms may be concerning from a public health perspective, as the food options provided by these platforms are predominantly unhealthy (Brar & Minaker, 2021; Partridge et al., 2020), contributing to higher energy intake and increased body weight (Albalawi et al., 2022; Rosenheck, 2008). This expansion of online food outlets introduces new complexities in understanding how the food environment influences dietary behaviours and highlights gaps in knowledge about the direct impact of OFDS on diet quality.

These complexities may have been further accelerated by the onset of the COVID-19 pandemic, during which restrictions on dining out, social distancing measures, and lockdowns may have prompted a shift toward using online platforms, including OFDS and online grocery services, for food purchases (Gupta et al., 2024; Jia et al., 2024). However, the evidence regarding increased OFDS use during the pandemic was mixed and may vary by personal characteristics (Jia et al., 2024). A repeated cross-sectional study design can help determine how OFDS availability and its impact on dietary behaviors have evolved over time.

Besides extending the reach of physical outlets, OFDS platforms may leverage targeted marketing and user profiling to reach specific customers (Botelho et al., 2023), further shifting the dynamics of food availability and dietary behaviours. These changes necessitate an investigation into how physical and online food outlet availability interact to influence dietary behaviours. The growing convenience of OFDS platforms may reduce the impact of physical outlets on diet as consumers increasingly opt for online purchases. Consequently, the influence of both physical and online outlets on dietary behaviours is likely to evolve, particularly in settings where OFDS platforms become increasingly popular. Despite its importance, no study to date has examined the simultaneous availability of physical and online food outlets.

We used repeated cross sectional survey data to investigate associations between physical and online food outlet availability, independently and mutually adjusted, and OOH-related dietary behaviours in Great Britain (GB) in 2022. Second, we explored potential effect modification by *online* food outlet availability in the association between *physical* outlet availability and dietary behaviours. Third, we sought to understand how these associations may have evolved with the increasing use of online food outlets from 2019 to 2022 by repeating the same analyses using data from 2019.

## 2 Methods

We used data from the UK arm of the 2019 and 2022 International Food Policy Study (IFPS), an annual repeated cross-sectional survey that examines dietary patterns and policy-relevant behaviours in five countries (Hammond et al., 2022). IFPS participants are asked to provide their postcode, permitting us to measure the availability of physical and online food outlets in participants' home environments. We obtained information on physical food outlet availability from Ordnance Survey's Points of Interest (OSPOI) dataset, and used web scraping techniques to collect data on the number of food outlets on OFDS platforms providing delivery services to UK-IFPS participants' postcodes.

The IFPS received ethics clearance from a University of Waterloo Research Ethics Board (REB #30829); the University of Cambridge Humanities and Social Sciences Research Ethics Committee also approved the UK-component of the IFPS.

### 2.1 Participant recruitment

IFPS surveys are conducted online annually in November and December with adults aged 18–100 years recruited in Australia, Canada, Mexico, the UK, and the United States, from the Nielsen Consumer Insights Global Panel and their partner panels. Data for this study were based on 2019 and 2022 UK surveys only and those included were adults living in GB with valid home postcode data as OSPOI data were only available in this region (i.e. GB; the UK minus Northern Ireland).

Email and panellist dashboard application invitations with unique survey access links were shared with a random sample of adult panellists stratified for age group and sex at birth. Quotas for age and sex were applied to facilitate recruitment of a diverse sample that approximated the known proportion in the UK population. Eligible participants were invited to complete a web-based survey that included questions on eating out frequency and sources of food prepared OOH. Informed consent was obtained before participation. Respondents received remuneration in accordance with their panel's usual incentive structure (e.g., points-based or monetary rewards, chances to win prizes). To further ensure that the study sample represents the average UK population, we applied post-stratification sample weights constructed using a raking algorithm based on known population totals by age group, sex at birth, region, education, and ethnicity, scaled to align with the analytic sample size. A full description of the IFPS study methods can be found in the 2019 and 2022 IFPS Technical Reports (Hammond et al., 2022).

### 2.2 Dependent variables

Three OOH-related dietary behaviours were examined using the 2019 and 2022 UK-IFPS survey data: self-reported frequency of consuming meals prepared OOH, frequency of physical food outlet use and frequency of online food outlet use. Supplementary Fig. 1 displays the hypothesized relationship between food outlet availability measures and outcomes. The frequency of consuming meals prepared OOH (for breakfast, lunch, and/or dinner) was assessed with the question, ‘During the past 7 days, how many meals did you get that were prepared away from home in places such as restaurants, fast food or take-away places, food stands, or from vending machines?’ Responses were numeric (0–21 meals), ‘Don't know,’ or ‘Refuse to answer.’ The latter two responses were coded as missing.

We also included self-reported frequency of physical and online food outlet use due to their direct relationship with our food outlet availability measures. Participants who consumed at least one meal prepared OOH reported the locations of purchases using the question, ‘You said you had [#] meal(s) prepared outside the home in the past 7 days. How many of those meals were … ' with four options: 1) ‘Ordered directly from a restaurant and delivered to you’, 2) ‘Ordered using a food delivery service (e.g., Uber Eats, Just Eat, Deliveroo) and delivered to you’, 3) ‘Purchased in person at a restaurant/food outlet within 5 min of your home’, and 4) ‘Purchased in person at a restaurant/food outlet more than 5 min away from your home'.

For this study, we used the data of answering options 2 through 4 to code the following two dependent variables: frequency of online food outlet use (answering option 2; number of meals – 0–21) and frequency of physical food outlet use combining the number of meals purchased in person within 5 min and more than 5 min from home (answering options 3 and 4; number of meals – 0–21). One participant's physical food outlet use was recoded from 24 to 21 meals per week to reflect a maximum of three meals per day consumed over seven days. Participants who reported consuming no meals prepared OOH were coded as having 0 meals from OFDS platforms and 0 meals in person at a restaurant/food outlet.

### 2.3 Independent variables

#### 2.3.1 Neighbourhood OOH outlets

We obtained data on the physical addresses of food outlets in GB from the OSPOI dataset relating to December 2019 and December 2022. OSPOI data is a directory of public and privately-owned businesses, educational institutions, and recreational facilities in GB (E. L. Wilkins et al., 2017). We used data from OSPOI food outlet categories that mostly sell meals (e.g. restaurants) or that are likely found on OFDS platforms (e.g. cafes). Categories included were fast food and takeaway outlets (OSPOI category 01020018); fast food delivery services (01020019); fish and chip shops (01020020); restaurants (01020043); and cafes, snack bars, tea rooms (01020013).

We adapted a method used by Wilkins et al. to deduplicate food outlet records in the OSPOI data (E. L. Wilkins et al., 2017). Duplicate entries were identified based on food outlet names and geographic coordinates, with allowances made for business name variations using string distance. This process excluded approximately 12% of business records within selected food outlet categories in 2019 and 2022.

Physical availability of food outlets was operationalised as the number of OOH outlets within a 1600m (approximately 1 mile) Euclidean buffer centred on participants' home postcode (i.e. neighbourhood OOH availability). This measure was chosen over other measures, such as proximity or relative density, due to its similarity with our online food outlet measure that captures the number of food outlets on OFDS platforms willing to deliver to participants’ home postcodes. This buffer size has also been demonstrated to relate closely to actual food purchasing behaviours among UK adults (Smith et al., 2010) and has been frequently used in previous UK studies (Burgoine et al., 2014, 2018). Nevertheless, to reflect the current uncertainty in food environment research regarding optimal buffer sizes, we also included 400m and 800m buffer sizes as sensitivity analysis with 2022 data (Hobbs et al., 2023).

#### 2.3.2 Outlets on OFDS platforms

We gathered online food outlet availability (i.e. OFDS availability) information intended to align with the 2022 IFPS data in March 2023 by employing web scraping techniques using Python and a web browser extension, in line with our previous research (Hoenink, Huang, et al., 2023). We operationalised OFDS availability as the number of food outlets on three OFDS platforms that provided delivery services to participants' full home postcode (e.g. CB1 3 PF). The OFDS platforms we included were Just Eat, Uber Eats, and Deliveroo, which held market shares of 31%, 22% and 19%, respectively, for meal delivery in the UK as of November 2022 (Statista, 2024).

We summed the number of food outlets from the three OFDS platforms as this represents the upper end of possible online food outlet availability. We did not deduplicate outlets across platforms, as doing so would not accurately reflect the practical availability of online food options. Deduplication would only capture unique outlets, whereas our approach accounts for the total number of outlets accessible to consumers across multiple platforms. As sensitivity analyses, we also included OFDS availability from single platforms, representing the lower limit of availability. Because in our outcome measure we focussed on food outlets selling meals prepared OOH from a restaurant/takeaway (and not e.g. ready-made meals offered at supermarkets), we excluded all popular supermarket chains operating on OFDS platforms (e.g. Sainsbury's, Tesco and ASDA). All other food outlets found on OFDS platforms were included in the analysis.

Since it is not possible to web scrape retrospectively, we used data previously scraped in November 2019 to align with the 2019 IFPS data (Hoenink, Huang, et al., 2023). This included the count of delivery options from Just Eat only, at the broader postcode district level (e.g. CB1; which is less accurate than at postcode unit level) and only for England instead of GB. As the 2019 online food outlet availability measure differed from that of 2022, we also replicated this 2019 method using 2022 data to ensure a degree of comparability between time periods.

### 2.4 Covariates

Sex at birth (male or female), age (continuous), ethnicity (majority (White) or minority (others)), educational level (‘low’ (high school completion or lower), ‘medium’ (some post-high school qualifications), and ‘high’ (university degree or higher)), number of children in the household and perceived income adequacy were included as individual-level covariates. Perceived income adequacy was measured using the question ‘Thinking about your total monthly income, how difficult or easy is it for you to make ends meet?’ (5-point likert scale ranging from very difficult to very easy). Due to data availability, number of children under 18 years in the household was only included in 2022 analyses.

We also considered the physical availability of supermarkets and measures of area-level deprivation. We hypothesized that adjusting for physical supermarkets in analyses with neighbourhood OOH outlets was necessary because both food outlets coexist in the same environment and directly compete for customers. In contrast, qualitative evidence suggests that online OOH outlets and supermarkets cater to different consumer needs and contexts, making their influences on dietary behaviours more distinct and less interdependent (Keeble et al., 2022). The determination of neighbourhood supermarket availability was akin to our approach for assessing neighbourhood OOH availability, sourced from the OSPOI datasets.

We used the latest Index of Multiple Deprivation (IMD) rankings from 2019 at the Lower-layer Super Output Area level as our measure of area-level deprivation (“Office for National Statistics [Internet]. National Statistics Postcode Lookup - 2021 Census. Available from: https://geoportal.statistics.gov.uk/datasets/ons::national-statistics-postcode-lookup-2021-census-august-2022-1/about. Accessed on March 2022,”). We used country-specific IMD measures for England, Scotland and Wales. IMD is a compound measure of relative deprivation, based on factors such as income, education and employment. We modelled deprivation as quartiles in each country as the calculation of deprivation varies.

### 2.5 Statistical analyses

We present weighted sample characteristics as the mean and standard deviation (SD) for normally distributed continuous variables and as the median and interquartile range (IQR) for skewed distributions. For categorical variables, we present frequencies and percentages (n (%)). Descriptive analyses compared neighbourhood OOH outlet availability to OFDS availability using Pearson correlation coefficients to assess their relationship.

First, to investigate the associations between neighbourhood OOH and OFDS availability—both independently and in combination—and OOH-related dietary behaviours, we used quasi-Poisson models suitable for overdispersed count data. While negative binomial models were considered as a better alternative than the quasi-Poisson models, these were incompatible with weighted estimates. Six models were developed, with two independent and three dependent variables. Complete case analyses were conducted, and weighted estimates are reported unless specified otherwise. To ensure comparability between food outlet availability measures given range differences, these were standardised by subtracting the mean.

All models were adjusted for age, sex, income adequacy, education level, ethnicity, presence of children, quintile of deprivation index, and supermarket availability (for neighbourhood OOH availability). After confirming no multicollinearity issues between the two availability measures using the variance inflation factor, models including both measures were also run.

Second, to explore potential effect modification by OFDS availability in the association between neighbourhood OOH availability and OOH-related dietary behaviours, we added interaction terms between the two availability measures in the quasi-Poisson models. Due to non-linear interactions, OFDS availability was categorised into quartiles, representing increasing availability. Statistically significant interaction terms (p < 0.05) prompted stratified analyses of neighbourhood OOH availability and outcomes by OFDS availability quartiles. Neighbourhood OOH availability was standardised separately within each quartile of OFDS availability.

Third, to understand how associations may have evolved with increasing use of OFDS platforms, we repeated cross-sectional analyses using 2019 data and replicated 2022 analyses with the same measurements and covariates (excluding presence of children in the household) as in 2019. For the first two objectives, we focused on 2022 analyses due to the enhanced availability and precision of the OFDS availability measure, which covered more OFDS platforms and more accurately defined locations across GB instead of just England. All analyses were conducted using R version 4.0.1.

## 3 Results

### 3.1 Sample characteristics

In 2019, data were available for 4139 UK-IFPS respondents. We excluded 1227 respondents due to missing postcode or outcome data, or because they lived outside England (where OFDS availability data were unavailable). The final analytical sample was 2912 respondents (70.4%) residing in England. In 2022, data were available for 4203 UK-IFPS participants. A total of 778 respondents were excluded due to missing postcode or outome data, or because they lived outside GB (n = 540; where neighbourhood OOH availability data were unavailable). The final analytical sample consisted of 3544 respondents (84.3%), with 3047 (72.5%) of them residing in England (when replicating 2019 analyses).

As shown in Table 1, in 2022, the majority of the weighted sample identified as White (86%) and had no children residing in their households (71%). The median frequency of consuming meals prepared OOH was 1 (IQR 0–3). The median frequency of physical and online food outlet use were 0 (IQR 0–1) and 1 (IQR 0–2), respectively. Sample characteristics of IFPS 2019 (N = 2912; only including those living in England) were mostly similar to that of 2022 except for the percentage of participants reporting to use physical and online food outlets; in 2022, 55% of participants reported using physical food outlets in the preceding 7 days and 25% of participants reported using online food outlets (data not shown in Table). In 2019, these percentages were 60% and 16%, respectively. Unweighted sociodemographic characteristics were similar to the weighted samples (Supplementary Table 1).

**Table 1:** Sociodemographic and meal purchasing characteristics among the 2019 England and 2022 Great Britain analytical IFPS weighted samples.

Sociodemographic and meal purchasing characteristics		2019 weighted sample (n = 2912)	2022 weighted sample (n = 3544)
**Age (years [mean, SD])**		50 (17)	50 (17)
**Sex (% female)**		1491 (51%)	1794 (51%)
**Ethnicity (% majority: White)**		2626 (90%)	3062 (86%)
**Children in household (%)**	0 children	N/A	2507 (71%)
	1 child	N/A	538 (15%)
	2 or more children	N/A	463 (13%)
**Education (%)**	Low: high school completion or lower	1493 (51%)	1427 (40%)
	Medium: some post-high school qualifications	620 (21%)	887 (25%)
	High: university degree or higher	818 (28%)	1230 (34%)
**Income adequacy (%)**	Very difficult	168 (6%)	264 (7%)
	Difficult	524 (18%)	814 (23%)
	Neither easy nor difficult	1037 (36%)	1319 (37%)
	Easy	746 (26%)	760 (21%)
	Very easy	455 (16%)	351 (10%)
**Deprivation index ranking (%)**	Q4: most deprived	833 (29%)	979 (28%)
	Q3	767 (26%)	869 (24%)
	Q2	693 (24%)	852 (24%)
	Q1: least deprived	637 (22%)	809 (23%)
**Number of neighbourhood OOH outlets in 1600m buffer (median (IQR))**		36 (14–80)	34 (11–83)
**Number of supermarkets in 1600m buffer (median (IQR))**		3 (1–4)	2 (1–4)
**Number of food outlets available from all three OFDS platforms (median (IQR))**		N/A	345 (91–867)
**Number of food outlets available from Just Eat (median (IQR)**		92 (38–188)	110 (34–236)
**Frequency of consuming meals prepared OOH in past 7 days (median (IQR))**		1 (0–3)	1 (0–3)
**Frequency of physical food outlet use in the past 7 days (median (IQR))**		1 (0–2)	1 (0–2)
**Frequency of online food outlet use in the past 7 days (median (IQR))**		0 (0–0)	0 (0–1)

Abbreviations: SD = Standard deviation, IQR = Interquartile range, N/A = Not applicable, OOH = Out-of-home, OFDS = Online Food Delivery Service.

Regarding food outlet availability, in 2022 the median number of neighbourhood OOH outlets was 33 (IQR 11–83) and the median number of food outlets on OFDS platforms was 345 (IQR 91–867; Table 1). The median number of food outlets on Deliveroo was highest, while Just Eat had the widest availability (only 2% of participants had no food outlets on Just Eat compared to 19% on Deliveroo; Supplementary Table 2). The correlation between food outlet availability measures increased with the expansion of neighbourhood OOH availability buffer zones (ranging from 0.35 to 0.76; Supplementary Table 3).

Due to data unavailability on delivery options from Deliveroo and Uber Eats for 2019, only delivery options from Just Eat at postcode district instead of postcode unit was included. Also, due to OFDS platform data availability, for 2019, only IFPS participants living in England were included.

### 3.2 Associations between availability measures and OOH-related dietary behaviours in 2022

**Fig. 1:**
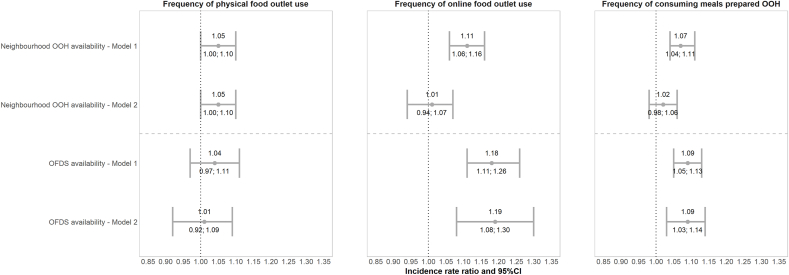
Incidence rate ratios and 95%CI of the association between neighbourhood OOH and OFDS availability, and OOH-related dietary behaviours in the Great Britain analytic IFPS weighted sample, 2022 (n = 3544). Neighbourhood OOH and OFDS availability measures were standardised by subtracting the mean to ensure comparability. Model 1 was adjusted for age, sex at birth, income adequacy, educational level, ethnicity, children in household, deprivation index, and supermarket availability (only for models with neighbourhood OOH availability due to their direct competition for customers, whereas we hypothesized that online OOH outlets and supermarkets cater to different consumer needs). Model 2 was additionally adjusted for the alternative food outlet availability measure. Abbreviations: OOH = out-of-home and OFDS = Online Food Delivery Service.

Greater neighbourhood OOH availability showed a trend towards being associated with frequency of physical food outlet use (IRR 1.05; 95%CI 1.00–1.10), whereas no association was found between OFDS availability and frequency of physical food outlet use (Fig. 1). Before adjustment for the alternative availability measure, both neighbourhood OOH and OFDS availability were associated with frequency of online food outlet use and consuming meals prepared OOH. However, in our adjusted models, only OFDS availability was associated with frequency of online food outlet use and consuming meals prepared OOH (Fig. 1; model 2). Specifically, a one standard deviation increase in OFDS availability was associated with a 19% (95%CI 8%–30%) increase in frequency of online food outlet use and a 9% (95%CI 3%–14%) increase in frequency of consuming meals prepared OOH. Findings with different neighbourhood buffers and single OFDS platforms were similar across all outcome measures (Supplementary Table 4).

### 3.3 Moderation by OFDS availability in 2022

**Fig. 2:**
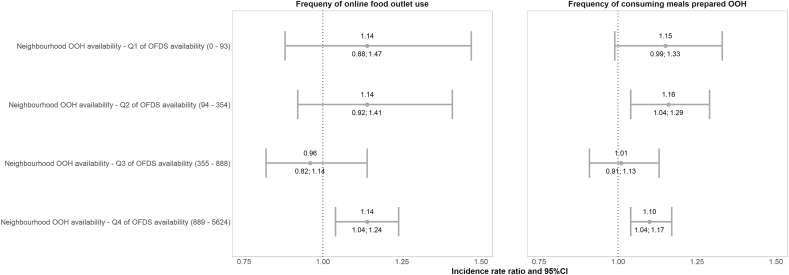
Incidence rate ratios and 95%CI of the association between neighbourhood OOH availability, and online food outlet use and frequency of consuming meals prepared OOH stratified by quartiles of OFDS availability in the Great Britain analytic IFPS weighted sample, 2022. There were n = 886 participants in each stratified sample and the neighbourhood OOH availability was standardised separately by each subgroup of OFDS availability quartile by subtracting the mean to ensure comparability. Models were adjusted for age, sex at birth, income adequacy, educational level, ethnicity, children in household, deprivation index and supermarket availability. Abbreviations: Q = quartile, OOH = out-of-home and OFDS = Online Food Delivery Service.

OFDS availability modified the associations between neighbourhood OOH availability and frequency of online food outlet use and consuming meals prepared OOH, but not frequency of physical food outlet use (data not shown). After stratifying analyses by quartiles of OFDS availability, a positive association between neighbourhood OOH availability and frequency of online food outlet use was observed only in areas with the highest OFDS availability (Q4) (Fig. 2). The association between neighbourhood OOH availability and frequency of consuming meals prepared OOH weakened from Q2 to Q3 of OFDS availability, but increased again in Q4. For example, a one standard deviation increase in neighbourhood OOH availability was associated with a 16% (95% CI 4%; 29%) greater frequency of consuming meals prepared OOH in Q2 of OFDS availability, and a 1% (95% CI -9%; 13%) increase in Q3.

Results varied slightly between analyses using neighbourhood OOH availability within 400m and 800m buffers (Supplementary Figs. 2 and 3). The association between neighbourhood OOH availability within 400m buffers and the frequency of consuming meals purchased OOH was observed only in the second quartile of OFDS availability. In contrast, no effect modification by OFDS availability was found in the association between neighbourhood OOH availability within 800m buffers and the frequency of consuming meals purchased OOH.

### 3.4 Changes between 2019 and 2022

In the 2019 analyses, data were available only for Just Eat in England (the market leading OFDS platform at that time), unlike the 2022 analyses, which included availability from all three OFDS platforms across GB. To ensure comparability, we repeated 2022 analyses using the same measures of availability as in 2019.

**Fig. 3:**
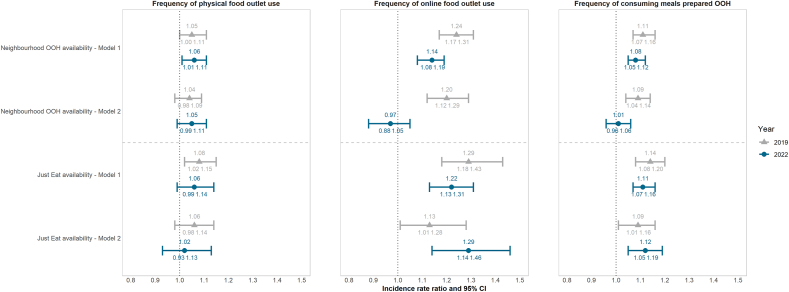
Incidence rate ratios and 95%CI of the association between neighbourhood OOH and OFDS availability, and OOH-related dietary behaviours in the England analytic IFPS weighted samples, 2019 (n = 2912) and 2022 (n = 3047). Neighbourhood OOH and OFDS availability measures were standardised by subtracting the mean to ensure comparability. Due to data availability, analyses were only conducted among those living in England. Model 1 was adjusted for age, sex at birth, income adequacy, educational level, ethnicity, deprivation index, and supermarket availability (only for models with neighbourhood OOH outlets due to their direct competition for customers, whereas online OOH outlets and supermarkets cater to different consumer needs). Model 2 was additionally adjusted for the alternative availability measure. Abbreviations: OOH = out-of-home.

While associations in 2019 mostly did not statistically significantly differ from those in 2022, as indicated by the overlapping 95% confidence intervals, some notable variations were observed (Fig. 3). In 2019, both neighbourhood OOH availability and Just Eat availability were associated with frequency of online food outlet use and consuming meals prepared OOH, even after adjusting for the other availability measure. For instance, a one standard deviation increase in neighbourhood OOH availability was associated with a 20% (95%CI 12%–29%) greater frequency of online food outlet use and a 9% (95%CI 4%–14%) greater frequency of consuming meals prepared OOH after adjusting for Just Eat availability. By 2022, only Just Eat availability was positively associated with frequency of online food outlet use and consuming meals prepared OOH after adjusting for neighbourhood OOH availability. These results were consistent with analyses using data from all three OFDS platforms (Fig. 1). Additionally, compared to 2019, the association between Just Eat availability and frequency of online food outlet use was stronger in 2022 after adjusting for neighbourhood OOH availability; although this difference was not statistically significant.

**Fig. 4:**
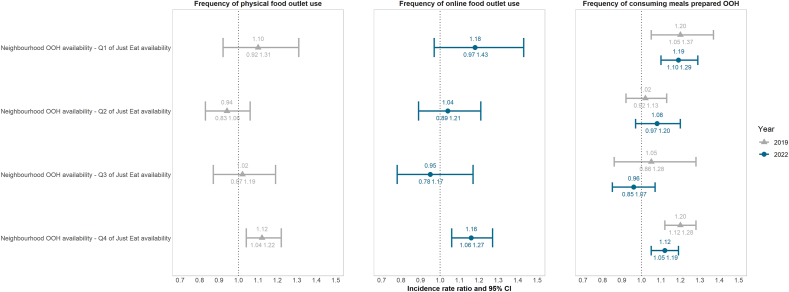
Incidence rate ratios and 95%CI of the association between neighbourhood OOH availability and dietary behaviours stratified by quartiles of OFDS availability in the England analytic IFPS weighted sample, 2019 and 2022. In 2019, there were n = 743, n = 726, n = 717 and n = 726 participants in Q1 through Q4, respectively. In 2022, this was n = 774, n = 755, n = 756 and n = 762. Due to data availability, analyses were only conducted among those living in England. Neighbourhood OOH availability was standardised separately for each subgroup of OFDS availability quartile by subtracting the mean to ensure comparability. Models were adjusted for age, sex at birth, income adequacy, educational level, ethnicity, deprivation index and supermarket availability. Abbreviations: Q = quartile and OOH = out-of-home.

In analyses investigating effect modification, we found that in 2019, Just Eat availability modified the association between neighbourhood OOH availability and frequency of *physical* food outlet use, but not *online* food outlet use (stratified analyses in Fig. 4). In 2022, the pattern was reversed: Just Eat availability modified the association between neighbourhood OOH availability and frequency of *online* food outlet use, but not *physical* food outlet use. Despite the different outcomes, the associations were consistent in 2019 and 2022; neighbourhood OOH availability was associated with frequency of physical outlet use (in 2019) and online food outlet use (in 2022) only in areas with the highest Just Eat delivery availability (Q4).

For the outcome frequency of consuming meals OOH, effect modification by Just Eat availability was similar in both 2019 and 2022. A pattern was observed where associations between neighbourhood OOH outlets and the frequency of consuming meals purchased OOH were present only in areas with the lowest and highest levels of Just Eat availability, suggesting a U-shaped trend. For example, in the highest quartile of Just Eat availability, a one standard deviation increase in neighbourhood OOH availability was associated with a 20% (95% CI: 12%–28%) greater frequency of consuming meals prepared OOH in 2019, and a 12% (95% CI: 5%–19%) increase in 2022. This U-shaped trend contrasts with the analyses that included data from all three OFDS platforms (Fig. 2), where similar effect sizes were observed in Q1 and Q2 of OFDS availability, but no association between neighbourhood OOH availability and frequency of consuming meals prepared OOH was found in Q1, while an association was present in Q2.

## 4 Discussion

In our study of adults living in GB, we investigated associations between neighbourhood OOH and OFDS availability in residential neighbourhoods and OOH-related dietary behaviours. In 2022, we found a trend suggesting an association between neighbourhood OOH availability and physical food outlet use. We also found that both neighbourhood OOH and OFDS availability measures were associated with online food outlet use and consuming meals prepared OOH. However, after adjusting for the alternative availability measure, only associations involving OFDS availability remained. Furthermore, OFDS availability modified associations between neighbourhood OOH availability and both online food outlet use and consuming meals prepared OOH, but not physical food outlet use. Stratified analyses indicated that the association between neighbourhood OOH availability and consuming meals prepared OOH weakened as OFDS availability increased. We found an increase in any online food outlet use in the preceding 7 days between 2019 and 2022, rising from 16% in 2019 to 25% in 2022. Comparing cross-sectional analyses from 2019 to 2022 suggested a shift in the association between food outlet availability and OOH-related dietary behaviours, highlighting a diminishing link between neighbourhood OOH availability and OOH-related dietary behaviours, contrasted with a strengthening link between OFDS availability and online food outlet use.

To our knowledge, this is the first study to examine both physical and online food outlet availability with respect to dietary behaviours, making direct comparisons with earlier research challenging. Similar to our study, previous research conducted before the rise in popularity of OFDS platforms have found evidence, albeit inconsistent, linking neighbourhood OOH availability to dietary outcomes (Caspi et al., 2012; Cobb et al., 2015). We found that the median number of food outlets on Just Eat in postcode districts grew from 92 in 2019 to 110 in 2022, similar to a broader trend of increasing OFDS availability observed in the UK from 2020 to 2021 (Kalbus et al., 2023).

Our findings consistently suggest a shift in purchasing behaviour where participants increasingly rely on OFDS platforms to purchase meals prepared OOH. Namely, initially, greater neighbourhood OOH outlet availability was linked to greater purchases of meals prepared OOH. However, this relationship disappeared when accounting for OFDS availability, which did remain positively associated with OOH meal consumption after adjusting for neighbourhood OOH availability. This suggests that individuals may be using neighbourhood outlets via online orders or opting for outlets outside their home environment (Hoenink, Eisink, et al., 2023).

Additionally, our effect modification analysis indicates that as OFDS availability increases, the influence of neighbourhood OOH availability on meal consumption weakens. Comparing data from 2019 to 2022 further underscores this shift: while both availability measures were positively associated with OOH meal consumption in 2019, by 2022, only OFDS availability was associated with OOH meal consumption. This trend highlights the growing importance of online food outlets, and suggests that this should be a focus for interventions and policy measures aimed at improving OOH meal consumption in the UK.

We found no evidence that the availability of OFDS outlets influences the relationship between neighbourhood OOH outlet availability and the frequency of physical food outlet use, likely due to the weak association between these factors. Additionally, findings suggest that food options on OFDS platforms are not linked to purchases at physical outlets. Similarly, neighbourhood dining options do not independently influence the decision to order food online. Instead, online food outlet use may be cued by factors such as convenience, a wider variety of options and promotional deals rather than the physical availability of food outlets (Keeble et al., 2022). Online and physical food outlets might serve different roles in our lives: we may visit physical outlets for special occasions, social gatherings, or work-related outings, where proximity to home is less relevant (Burgoine et al., 2014). In contrast, we may choose to order online when gathering at home or when we prefer not to go out (Keeble et al., 2022).

Our findings have implications for researchers studying the food environment in contexts where OFDS platform use is high (e.g. the UK, Switzerland and the Netherlands (Hoenink, Huang, et al., 2023)). They highlight the necessity of incorporating online food outlet measures in future research. Our observations suggest that recent UK research finding positive associations between neighbourhood OOH outlets and dietary behaviours may no longer reflect the current influence of these outlets, as the landscape has likely evolved since those studies were conducted. The stronger association between OFDS availability and consuming meals prepared OOH, compared to neighbourhood OOH availability, suggests a potential shift in dietary behaviours, highlighting the need for further exploration of its long-term public health impacts. Future research should extend our findings to other countries with different degrees of online food outlet use, such as Italy and Spain (Hoenink, Huang, et al., 2023), and in different contexts (e.g. the workplace).

### 4.1 Strengths and limitations

To our knowledge, this is the first study to examine the links of both physical and online food outlet availability on OOH-related dietary behaviours. Automated data collection enabled us to gather exposure and outcome data nearly simultaneously in 2022, within a few months, from a large adult sample. This approach helped minimize potential exposure misclassification. Recognizing that OFDS availability might vary by time of day and delivery driver availability, we conducted most of the web scraping on weekday evenings.

The IFPS survey was partly developed from existing measures used in national surveys known for their validity and reliability (e.g. the question assessing the frequency of consuming meals prepared OOH was adapted from NHANES) (Hammond et al., 2022). While respondents were recruited through nonprobability sampling, potentially limiting national representativeness (Keeble et al., 2021), we applied post-stratification sample weights to enhance representativeness. A limitation of this study is its focus solely on the residential neighbourhood environment versus e.g. activity spaces. The 1600m buffer used to define physical food outlet availability may not fully reflect participants’ actual food outlet use (Hoenink, Eisink, et al., 2023). Furthermore, there are some variations in the types of food outlets captured online versus physical availability measures; for instance, we included OSPOI categories that exclusively list food outlets while excluding categories that may encompass businesses selling both food and non-food products. Additionally, the cross-sectional design restricts our ability to draw causal inferences. The self-reported nature of the data, collected via online surveys, may have introduced social desirability bias, potentially leading to under-reporting of the frequency of consuming meals prepared OOH. However, completing the surveys online might have provided participants a sense of anonymity, possibly mitigating this bias (Krumpal, 2013).

Future research could use IFPS data from other countries to assess the generalisability of our results. Moreover, considering potential seasonal variations in the use of online food outlets, it would be valuable to replicate these analyses with data collected during other seasons, such as spring and summer. For example, colder weather or shorter daylight hours may increase the use of delivery services due to reduced willingness to travel to physical outlets. Understanding these seasonal dynamics could provide further insights into the temporal patterns of online food outlet use.

### 4.2 Conclusion

In our study of over 3500 participants living in Great Britain, we found that the link between online food outlet availability in residential neighbourhoods and dietary behaviours is becoming increasingly stronger. Traditional food environment research focusing solely on the physical availability of food outlets around the home may not fully capture the reality of food outlet availability and its impact on dietary behaviours, particularly in contexts where online food outlets are prevalent. To effectively address poor diet and related non-communicable diseases, policy measures should consider online food environments. Such an approach will ensure that interventions are aligned with the evolving landscape of food access, ultimately promoting healthier population-wide dietary health.

## CRediT authorship contribution statement

**Jody C. Hoenink:** Writing – original draft, Methodology, Investigation, Formal analysis, Conceptualization. **Yuru Huang:** Writing – review & editing, Data curation. **Matthew Keeble:** Writing – review & editing, Data curation. **Joreintje D. Mackenbach:** Writing – review & editing. **Maria G.M. de Pinho:** Writing – review & editing. **Lana Vanderlee:** Writing – review & editing, Funding acquisition. **David Hammond:** Writing – review & editing, Funding acquisition. **Christine M. White:** Writing – review & editing, Data curation. **Thomas Burgoine:** Writing – review & editing, Supervision. **Jean Adams:** Writing – review & editing, Supervision, Methodology.

## Consent for publication

Not applicable.

## Availability of data and materials

The datasets analysed during the current study are available from the corresponding author on reasonable request. The original protocol is available from the corresponding author on request. Details of the study materials and data can be found on the International Food Policy website (https://foodpolicystudy.com/about/).

## Ethics approval and consent to participate

The study complies with the Declaration of Helsinki and was approved by the University of Waterloo Research Ethics Board (REB #30829; IFPS) and the University of Cambridge Humanities and Social Sciences Research Ethics Committee (UK-IFPS). Informed consent was obtained from all the participants before starting the study.

## Declaration of generative AI and AI-assisted technologies in the writing process

During the preparation of this work the author(s) used ChatGPT4o in order to improve readability and language of the manuscript. After using this tool, the authors reviewed and edited the content as needed and take full responsibility for the content of the published article.

## Funding

The International Food Policy Study was funded by a Project Grant from the Canadian Institutes of Health Research (CIHR) (PJT-162167). JCH, TB and JA were supported by the Medical Research Council [Unit Programme number MC_UU_00006/7]. JDM and MGMP were funded through the EXPANSE project, which received funding from the European Union's Horizon 2020 research and innovation program under grant agreement number 874627. The funders played no role in the design of the study, the collection, analysis, and interpretation of data, or the writing of the manuscript. For the purpose of Open Access, the authors have applied a Creative Commons Attribution (CC BY) licence to any Author Accepted Manuscript version arising.

## Declaration of competing interest

DH has provided paid expert testimony on behalf of public health authorities in response to legal challenges from the food and beverage industry. The other authors declare that they have no known competing financial interests or personal relationships that could have appeared to influence the work reported in this manuscript.

## Data Availability

Data will be made available on request.
