# Supplementary material for: Physical and online food outlet availability and its influence on out-of-home dietary behaviours in Great Britain: A repeated cross-sectional study
Source: SSM Popul Health. 2025 Mar 5;30:101773. doi: 10.1016/j.ssmph.2025.101773 (PMC11932679; doi:10.1016/j.ssmph.2025.101773)
Supplement: Multimedia component 1 [file mmc1.docx]

# Supplementary Material


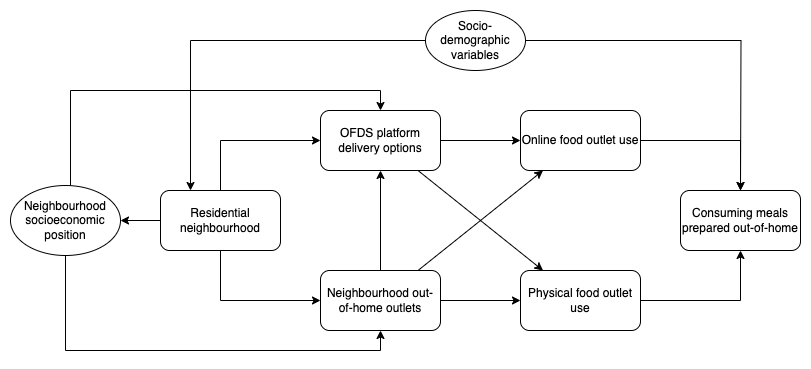


Supplementary Figure 1. Hypothesised pathway through which residential food outlet availability influences the consumption of meals prepared OOH.

Supplementary Table 1. Sociodemographic and meal purchasing characteristics among the 2019 England and 2022 Great Britain analytical IFPS unweighted samples

| **Sociodemographic and meal purchasing characteristics** | | **Unweighted sample 2019 (n=2912)** | **Unweighted sample 2022 (n=3544)** |
| --- | --- | --- | --- |
| **Age (mean, SD)** | | 51 (17) | 50 (17) |
| **Sex (% female)** | | 1456 (50%) | 1781 (50%) |
| **Ethnicity (% majority: white)** | | 2661 (91%) | 3162 (89%) |
| **Children in**  **household (%)** | 0 children | NA | 2518 (71%) |
|  | 1 child | NA | 554 (16%) |
|  | 2 or more children | NA | 472 (13%) |
| **Education (%)** | Low: high school completion or lower | 858 (29%) | 1010 (29%) |
|  | Medium: some post-high school qualifications | 771 (26%) | 899 (25%) |
|  | High: university degree or higher | 1283 (44%) | 1635 (46%) |
| **Income**  **adequacy (%)** | Very difficult | 143 (5%) | 239 (7%) |
|  | Difficult | 452 (16%) | 762 (21%) |
|  | Neither easy nor difficult | 991 (34%) | 1320 (37%) |
|  | Easy | 807 (28%) | 828 (23%) |
|  | Very easy | 519 (18%) | 395 (11%) |
| **Deprivation index ranking (%)** | Q4: most deprived | 730 (25%) | 888 (25%) |
|  | Q3 | 727 (25%) | 885 (25%) |
|  | Q2 | 727 (25%) | 885 (25%) |
|  | Q1: least deprived | 728 (25%) | 886 (25%) |
| **Number of neighbourhood OOH outlets in 1600m buffer (median (IQR))** | | 34 (13 – 80) | 0 (0 – 0) |
| **Number of supermarkets in 1600m buffer (median (IQR))** | | 3 (1 – 4) | 2 (1 – 4) |
| **Number of food outlets available on all three OFDS platforms (median (IQR))** | | NA | 354 (94 – 888) |
| **Number of food outlets available from Just Eat (median (IQR)** | | 89 (37 – 177) | 110 (34 – 241) |
| **Frequency of consuming meals prepared OOH**  **in past 7 days (median (IQR))** | | 1 (0 – 3) | 1 (0 – 3) |
| **Frequency of physical food outlet use in the past 7 days (median (IQR))** | | 1 (0 – 1) | 1 (0 – 2) |
| **Frequency of online food outlet use in the past 7 days (median (IQR))** | | 0 (0 – 0) | 0 (0 – 1) |

Abbreviations: SD = Standard deviation, IQR = Interquartile range, NA = Not applicable, OOH = Out-of-home, OFDS = Online Food Delivery Service

Due to missing data on delivery options from Deliveroo and Uber Eats for 2019, only delivery options from Just Eat at postcode district instead of postcode unit was included.

Supplementary Table 2. Descriptive statistics of food outlet availability measures among the Great Britain analytic IFPS unweighted sample, 2022 (n=3544)

|  | Neighbourhood OOH availability in 400m buffer | Neighbourhood OOH availability in 800m buffer | Neighbourhood OOH availability in 1600m buffer | Food outlet availability from all three platforms | Food outlet availability from Just Eat | Food outlet availability from Uber Eats | Food outlet availability from Deliveroo |
| --- | --- | --- | --- | --- | --- | --- | --- |
| Median (IQR) | 1 (0 – 6) | 8 (2 – 26) | 35 (12 – 84) | 354 (93 – 888) | 115 (40 – 234) | 78 (23 – 226) | 146 (27 – 422) |
| Count of 0 neighbourhood OOH outlets or OFDS outlets (%) | 1367 (39%) | 499 (14%) | 239 (7%) | 66 (2%) | 67 (2%) | 341 (10%) | 664 (19%) |
| Median food outlet availability in areas with no neighbourhood OOH availability in 1600m buffers (IQR) | - | - | - | 11 (3 – 54) | 8 (3 – 26) | 1 ( 0 – 13) | 0 ( 0 – 6) |
| Median food outlet availability in areas with no Deliveroo availability (%) | 0 (0 – 2) | 1 (0 – 6) | 3 (0 – 15) | - | - | - | - |

Abbreviations: IQR = Interquartile range, OOH = out-of-home, OFDS = Online Food Delivery Service

Supplementary Table 3. Correlation matrix of online and physical food outlet availability in Great Britain analytic IFPS unweighted sample, 2022 (n=3544)

|  | Neighbour-hood OOH availability in 400m buffer | Neighbour-hood OOH availability in 800m buffer | Neighbour-hood OOH availability in 1600m buffer | Food outlet availability from all three platforms | Food outlet availability from Just Eat | Food outlet availability from Uber Eats | Food outlet availability from Deliveroo |
| --- | --- | --- | --- | --- | --- | --- | --- |
| Neighbourhood OOH availability in 400m buffer | 1.00 | 0.91 | 0.75 | 0.46 | 0.50 | 0.35 | 0.45 |
| Neighbourhood OOH availability in 800m buffer | - | 1.00 | 0.90 | 0.56 | 0.61 | 0.42 | 0.55 |
| Neighbourhood OOH availability in 1600m buffer | - | - | 1.00 | 0.69 | 0.76 | 0.51 | 0.69 |
| Food outlet availability from all three platforms | - | - | - | 1.00 | 0.95 | 0.92 | 0.99 |
| Food outlet availability from Just Eat | - | - | - | - | 1.00 | 0.84 | 0.91 |
| Food outlet availability from Uber Eats | - | - | - | - | - | 1.00 | 0.86 |
| Food outlet availability from Deliveroo | - | - | - | - | - | - | 1.00 |

Abbreviations: OOH = out-of-home

Supplementary Table 4. Incidence rate ratios and 95%CI of the association between neighbourhood OOH and OFDS availability, and OOH-related dietary behaviours in Great Britain analytic IFPS weighted sample, 2022 (n=3544)

|  | **Frequency of physical food outlet use** | | | | **Frequency of online food**  **outlet use** | | | | **Frequency of consuming meals prepared OOH** | | | |
| --- | --- | --- | --- | --- | --- | --- | --- | --- | --- | --- | --- | --- |
|  | Model 1 | | Model 2 | | Model 1 | | Model 2 | | Model 1 | | Model 2 | |
|  | IRR | 95%CI | IRR | 95%CI | IRR | 95%CI | IRR | 95%CI | IRR | 95%CI | IRR | 95%CI |
| Neighbourhood OOH availability in 400m buffer | **1.05** | **1.01; 1.09** | 1.04 | 1.00; 1.09 | **1.06** | **1.02; 1.10** | 1.00 | 0.94; 1.05 | **1.05** | **1.02; 1.08** | 1.03 | 0.99; 1.06 |
| Neighbourhood OOH availability in 800m buffer | **1.06** | **1.02; 1.10** | **1.06** | **1.01; 1.11** | **1.08** | **1.03; 1.12** | 1.00 | 0.94; 1.05 | **1.07** | **1.04; 1.10** | **1.03** | **1.00; 1.07** |
| Food outlet availability from Just Eat | 1.05 | 0.98; 1.12 | 1.01 | 0.92; 1.12 | **1.19** | **1.11; 1.26** | **1.25** | **1.12; 1.39** | **1.09** | **1.05; 1.13** | **1.09** | **1.03; 1.16** |
| Food outlet availability from Uber Eats | 1.02 | 0.96; 1.08 | 0.99 | 0.93; 1.06 | **1.18** | **1.09; 1.28** | **1.16** | **1.06; 1.27** | **1.09** | **1.05; 1.14** | **1.05** | **1.01; 1.08** |
| Food outlet availability from Deliveroo | 1.04 | 0.97; 1.11 | 1.01 | 0.93; 1.10 | **1.16** | **1.09; 1.23** | **1.15** | **1.06; 1.25** | **1.08** | **1.05; 1.12** | **1.07** | **1.03; 1.12** |

Abbreviations: IRR = Incidence Rate Ratio, 95%CI = 95% Confidence Interval, OOH = out-of-home

Neighbourhood OOH and OFDS availability measures were standardised by subtracting the mean to ensure comparability

Bold values are statistically significant after adjustment for multiple testing

Model 1 is adjusted for age, sex, income adequacy, educational level, ethnicity, children in household, deprivation index, and supermarket availability (only for models with neighbourhood OOH availability measures)

Model 2 is additionally adjusted for the alternative food outlet availability variable (neighbourhood OOH availability in 1600m buffers or OFDS availability from all three OFDS platforms)


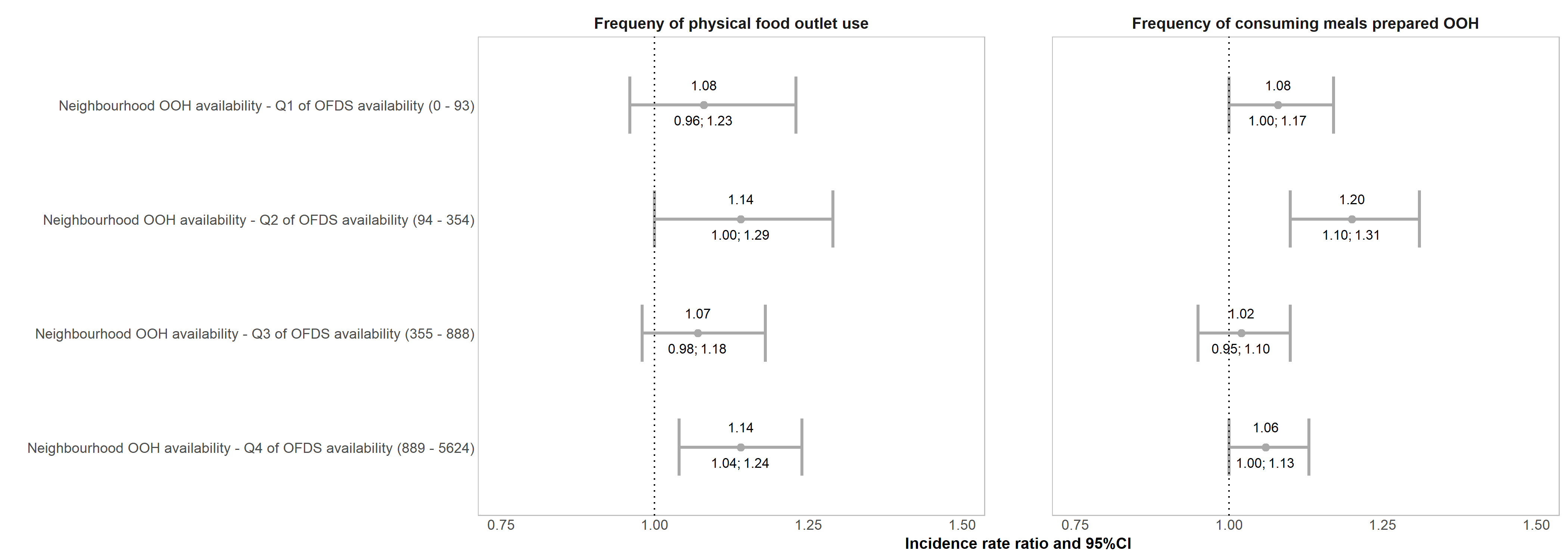


Supplementary Figure 2. Incidence rate ratios and 95%CI of the association between neighbourhood OOH availability in 400m buffers, and frequency of online food outlet use and OOH-prepared meal consumption stratified by quartiles of OFDS availability in Great Britain analytic IFPS weighted sample, 2022. There were n=886 participants in each stratified sample and neighbourhood OOH availability was standardised separately by each subgroup of OFDS availability quartile by subtracting the mean to ensure comparability. Models were adjusted for age, sex at birth, income adequacy, educational level, ethnicity, children in household, deprivation index and supermarket availability. Abbreviations: Q = quartile, OOH = out-of-home and OFDS = Online Food Delivery Service.


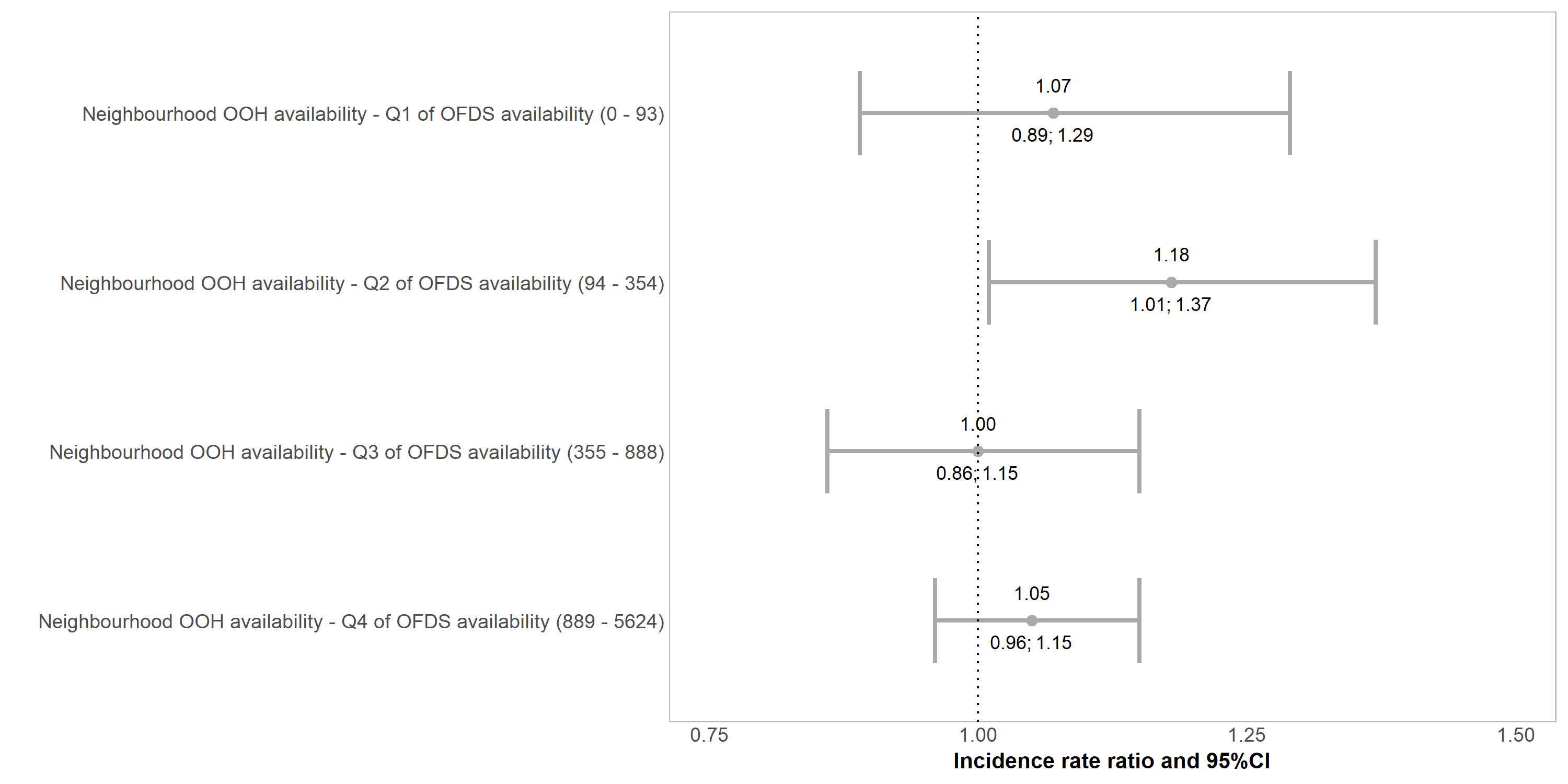


Supplementary Figure 3. Incidence rate ratios and 95%CI of the association between neighbourhood OOH availability in 800m buffers and frequency of online food outlet use stratified by quartiles of OFDS availability in Great Britain analytic IFPS weighted sample, 2022. There were n=886 participants in each stratified sample and neighbourhood OOH availability was standardised separately by each subgroup of OFDS availability quartile by subtracting the mean to ensure comparability. Models were adjusted for age, sex at birth, income adequacy, educational level, ethnicity, children in household, deprivation index and supermarket availability. Abbreviations: Q = quartile, OOH = out-of-home and OFDS = Online Food Delivery Service.
